# Estimating foliar anthocyanin content of purple corn via hyperspectral model

**DOI:** 10.1002/fsn3.588

**Published:** 2018-02-04

**Authors:** Xiaohe Gu, Wenqian Cai, Youbo Fan, Yue Ma, Xiaoyan Zhao, Chao Zhang

**Affiliations:** ^1^ Beijing Academy of Agriculture and Forestry Sciences Beijing Research Center for Information Technology in Agriculture Beijing China; ^2^ Beijing Academy of Agriculture and Forestry Sciences Beijing Vegetable Research Center Beijing Key Laboratory of Fruits and Vegetable Storage and Processing Key Laboratory of Vegetable Postharvest Processing Ministry of Agriculture Beijing China

**Keywords:** anthocyanin content, hyperspectral model, pH differential method, purple corn, sensitive band

## Abstract

To date, the foliar anthocyanin content was either determined via the pH differential or HPLC methods, both of which are slow and destructive. Here, a hyperspectral model was established to estimate the foliar anthocyanin content of purple corn (*Zea mays* L. var. Jingzi No. 1). The reflectivity (*P*) of the foliar hyperspectral was inverted to 1/*P*, lg *P*, 1/lg *P*, ${\left(P\right)}^{\prime }$, ${\left(1/P\right)}^{\prime }$, ${\left(\mathrm{lg}P\right)}^{\prime }$, and ${\left(1/\mathrm{lg}P\right)}^{\prime }$. The correlation coefficient between these inversions and the foliar anthocyanin content was plotted against the hyperspectral wavelength. The wavelength of inversions around 650 nm was sensitive to the foliar anthocyanin content. The hyperspectral model was fitted via linear, polynomial, power, exponential, and logarithmic functions with the sensitive band as independent variable and the anthocyanin content as function. The hyperspectral model (*y* = 3,000,000,000 × *W*_685_^4.5896^) fitted via inversion of ${\left(\mathrm{lg}P\right)}^{\prime }$ showed the highest determination coefficients (0.768) among all models. The hyperspectral model was well validated with a determination coefficient of 0.932 and an RMSE of 0.0065. Moreover, the accuracy and stability of the hyperspectral model were further enhanced with a determination coefficient of 0.954 and RMSE of 0.0047 when the anthocyanin content of the sample was below 20 mg/g. Hence, the hyperspectral model estimated the foliar anthocyanin content of purple corn quickly and nondestructively.

## 1 INTRODUCTION

**Figure 1:**
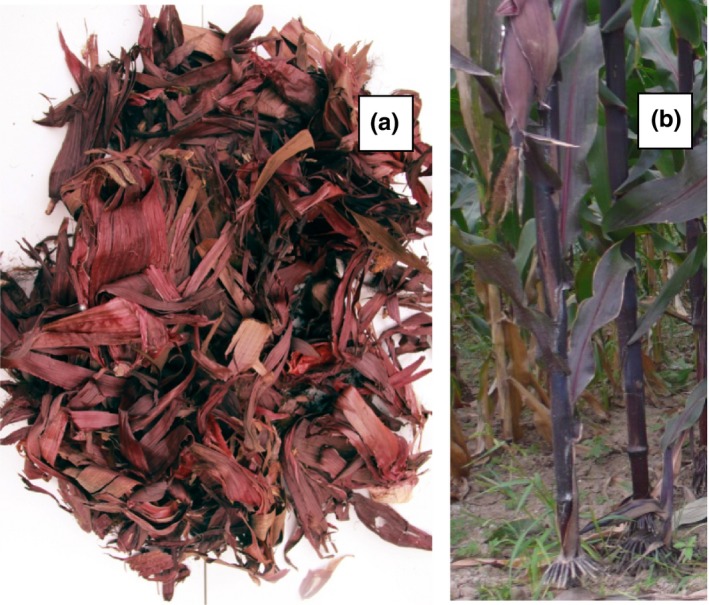
Purple corn leaf (a) and whole purple corn plant (b)

Anthocyanins are a group of water‐soluble pigments with a 2‐phenylbenzophyrylium (flavylium) structure, which not only provide vivid colors, but also present many physiological functions, such as antioxidant, antiobesity, antimutagenic, and anticarcinogenic capacities (Abdel‐Aal, Young, & Rabalski, 2006; Kähkönen & Heinonen, 2003; Katsube, Iwashita, Tsushida, Yamaki, & Kobori, 2003; Tsuda, Horio, Uchida, Aoki, & Osawa, 2003). Jingzi No. 1 (*Zea mays* L.) is a newly bred variety of purple corn. The entire plant of Jingzi No. 1 is dark red and rich in anthocyanins (Figure 1), which makes it a good resource for anthocyanin extraction (Aoki, Kuze, & Kato, 2002). Remarkably, the anthocyanin content of the plant increased with the growth after pollination, but then decreased. Consequently, the proper harvest time became the key for anthocyanin extraction. Traditionally, the anthocyanin content is either determined via pH differential or HPLC methods (Zhao, Corrales, Zhang, Hu, & Ma, 2008). Both methods destroy the sample and extract the anthocyanin before determination. Specifically, the pH differential method typically takes 2–3 hr for anthocyanin extraction and 10 min for the spectroscopic measurement, while the HPLC method takes 2–3 hr for anthocyanin extraction and 10–60 min for HPLC elution.

In contrast, a hyperspectrum technique is a nondestructive, quick, and simple method to measure the pigment of plants (Palmer et al., 2006; Qin & Lu, 2008). The technique measures the foliar nitrogen content (He et al., 2016), chlorophyll content (Croft et al., 2015; Zhang, Chen, Miller, & Noland, 2008), and moisture content (Clevers, Kooistra, & Schaepman, 2010). However, the hyperspectral model of the anthocyanin content was not plotted due to a weak relationship between the foliar anthocyanin content and the hyperspectrum. One possible reason for this was the overlap between the chlorophyll and anthocyanin absorption peaks (Sims & Gamon, 2002). The other reason was the influence of the moisture and leaf scattering absorption (Hatfield, Gitelson, Schepers, & Walthall, 2008).

Therefore, a hyperspectral model was developed to evaluate the foliar anthocyanin content of Jingzi No. 1. To reduce the influence of chlorophyll and moisture as well as leaf scattering, a sensitive band was selected via multiple linear regressions. Then, the hyperspectral model was inversed via linear, polynomial, power, exponential, and logarithmic functions with the sensitive band as independent variable and the anthocyanin content as function. The hyperspectral model will provide nondestructive and quick support for the harvest of purple corn. Moreover, the further application of the hyperspectral model based on the geographic information will provide instant and reliable support for the harvest decision at large scale.

## 2 MATERIALS AND METHODS

### 2.1 Foliar sampling

Jingzi No. 1 (*Zea mays* L.) was harvested 85 days after sowing in September 2014 and 2015 in Yanqing Farmer. The whole leaf was dark red with a moisture content of 6%–12%. A piece of leaf was randomly picked from a strain of the corn and was cut into an 8 cm × 6 cm blade in the middle. The cut leaf was immediately scanned via mobile hyperspectral radiometer (ASD Field Spec Pro FR, US), which was coupled with a LI‐Cor1800‐12S external integrating sphere. After calibration with the white board, the hyperspectrum was collected from 350 to 2,500 nm at an interval of 1 nm. The hyperspectrum was scanned with a distance between sample and radiometer of 5 cm and a view angle of 25° on a sunny and clear day from 10:00 a.m. to 2:00 p.m. The foliar spectral was repeated 10 times, and the obtained results were averaged. The external integrating sphere was used to ensure the repeatability of the spectra. The data were processed with the software ViewSpec Pro, Version 2.14 (Analytical Spectral Device, Inc5335 Sterling Drive Suite A, Boulder, CO 80301). The scanned leaf was numbered and sealed in a polyethylene bag for anthocyanin content determination in the laboratory. A total of 500 pieces of leaves were collected.

### 2.2 Measurement of pH differential method

The anthocyanin content of samples was determined via pH differential method previously described (Zhao et al., 2008). The leaf (10 g) was smashed and stirred in 50 ml liquid (a solution of 60% (v/v) ethanol acidified with citric acid (1%, w/v)) at 60°C for 120 min. The ethanol extracts were centrifuged at 9,000 rpm and 20°C for 10 min. The supernatants were evaporated to dryness at 46°C with a rotary evaporator Büchi R‐3000 (Büchi Labortechnik AG, Switzerland). Then, the concentrate was freeze‐dried. An aliquot of the dried concentrate (1 mg) was placed into a 25‐ml volumetric flask and filled to the final volume with pH 1.0 buffer. Another 1 mg of the sample was placed into a 25‐ml volumetric flask and filled to a final volume with pH 4.5 buffer. Absorbance was measured via spectrophotometer (UV‐1800, Shimadzu, Japan) at 510 and 700 nm, respectively. Absorbance was calculated as Abs = (*A*_510 nm_ − *A*_700 nm_) pH_1.0_ − (*A*_510 nm_ − *A*_700 nm_) pH_4.5_ with the molar extinction coefficient for cyanidin 3‐glucoside of 26,900. Total anthocyanin content was calculated using the following equation and expressed as grams of cyanidin 3‐glucoside equivalents per 1 g sample (Equation (1)).

(1) $$\text{Anthocyanin content (mg/g)}=\frac{\text{Abs}}{\mathrm{eL}}\times \text{MW}\times D\times \frac{V}{G}$$

where Abs represents the absorbance, *e* represents the cyanidin 3‐glucoside molar absorbance [26,900 ml/(mmol·cm)], *L* represents the cell path length (1 cm), MW represents the molecular weight of anthocyanin (449.2 Da), *D* is a dilution factor, *V* represents the final volume (ml), and *G* represents the dry material (mg).

### 2.3 Screening of the sensitive band

The reflectivity of the hyperspectrum (*P*) was inverted to the reciprocal of the reflectivity (1/*P*), the logarithm of the reflectivity (lg *P*), the reciprocal of the logarithm of the reflectivity (1/lg *P*), the first‐order differential of the reflectivity ${\left(P\right)}^{\prime }$, the first‐order differential of the reciprocal of the reflectivity ${\left(1/P\right)}^{\prime }$, the first‐order differential of the logarithm of the reflectivity (${\left(\mathrm{lg}P\right)}^{\prime }$), and the first‐order differential of the reciprocal of the logarithm of the reflectivity ${\left(1/\mathrm{lg}P\right)}^{\prime }.$

The differential inversion of spectral reflectance was calculated via Equation (2).

(2) $${\left(P\right)}^{\prime }=\left[P\left(\lambda_{i}\right)-P\left(\lambda_{i-1}\right)\right]/2\Delta \lambda$$

where *P* represents the reflectance of a band of λ and Δλ represents the interval from λ_*i*_ to λ_*i*−1_.

The correlation coefficient between the foliar anthocyanin content and hyperspectral vectors or the inverted vectors was evaluated via Equation (3). The sensitive bands were selected depending on the correlation coefficient.

(3) $$R=\frac{\sum_{n=0}^{N}\left(P_{\mathrm{ni}}-{\overset{¯}{P}}_{i}\right)\left({\mathrm{LAC}}_{n}-\bar{\mathrm{LAC}}\right)}{\sqrt{\sum_{n=1}^{N}{\left(P_{\mathrm{ni}}-{\overset{¯}{P}}_{i}\right)}^{2}\sum_{n=1}^{N}{\left({\mathrm{LAC}}_{n}-\bar{\mathrm{LAC}}\right)}^{2}}}$$

where *P*_ni_ represents the reflectivity or its transforms of No. *i* band *i* of No. *n* leaf sample, ${\overset{¯}{P}}_{i}$ represents the average reflectivity or its transforms of No. *i* of all leaf samples, LAC_*n*_ represents the anthocyanin content of No. *n* leaf sample, $\overset{¯}{\text{LAC}}$ represents the measured average anthocyanin content of all purple leaf samples, and *N* represents the number of all samples.

### 2.4 Establishment and validation of the hyperspectral model

The hyperspectral model for the anthocyanin content was fitted via linear, polynomial, power, exponential, and logarithmic functions with the sensitive band as independent variable and the anthocyanin content as function. Specifically, the reflectivity value of the sensitive band was plotted against the foliar anthocyanin content via linear (*Y* = *a* × *X* + *b*), polynomial (*Y* = *a* × *X* ^2^ + *b* × *X* + *c*), power (*Y* = *a* × *X* ^*b*^), exponential (*Y* = *a* × *e* ^*b*×*X*^), and logarithmic (*Y* = *a* × Ln(*X*) + *b*) functions. A total of 400 samples were used to train the hyperspectral model, and the remaining 100 samples were used to validate the accuracy via both the determination coefficient (*R* ^2^) and the root mean square error (RMSE, Equation (4)).

(4) $$\text{RMSE}=\sqrt{\frac{\sum_{i=1}^{N}{\left({\text{LAC}}_{i}-{\text{PLAC}}_{i}\right)}^{2}}{N}}$$

where LAC_*i*_ and PLAC_*i*_ represent the anthocyanin content and predicted anthocyanin content of the purple corn leaf, respectively; *N* represents the number of the validation.

## 3 RESULTS AND DISCUSSION

### 3.1 Screening of sensitive bands

Hyperspectrals usually contain noise due to atmospheric, instrumental, and geometric distortions (Gomez, Oltra‐Carrió, Bacha, Lagacherie, & Briottet, 2015). Consequently, reducing the atmospheric influences and shortening the hyperspectrum range reduced the noises of the hyperspectral. Specifically, the LI‐C or 1800‐12S external integrating sphere was coupled with the hyperspectral radiometer. The external integrating sphere provided stable illumination and appropriate reflection for the sample, thus reducing the noise of the reflectivity. Moreover, moisture is another factor that enhanced the noise of the reflectivity (Croft et al., 2015; Zhang et al., 2008). The hyperspectrum of 1,400~2,500 nm is sensitive to the moisture content, especially to the bands of 1,450 and 1940 nm (Clevers et al., 2010). Consequently, only the hyperspectrum of 400~1,400 nm was used in the following inversions to reduce noise.

Prof. Zhou, a reviewer, suggested that the reflectivity of chlorophyll will overlap with that of anthocyanin, and the moisture and some other factors would affect the determination coefficient and RMSE of the model. A nonlinear model could remove the effect of the foliar chlorophyll and moisture. His groups used a nonlinear model to establish the relationship between the anthocyanin/chlorophyll content and reflectance of 400–750 nm spectrum and excluded the influence of atmospheric environments. The nonlinear model is well‐predicted anthocyanin and chlorophyll content in grapevine leaves (Qin, 2011; Qin, Rundquist, Gitelson, Tan, & Steele, 2010). Hence, a nonlinear model could be a better model to predict the foliar anthocyanin content in purple corn. We carefully evaluated the foliar anthocyanin, chlorophyll, and moisture of purple corns. Being different to the grape leaves, purple corn leaves were rich in anthocyanins but lack of chlorophyll and moisture. The foliar anthocyanin content ranged from 0.09 to 44.3 mg/g with an average value of 17.8 mg/g, while the foliar chlorophyll content was 0.056 ± 0.026 mg/g. The foliar anthocyanin content was about 300 times higher than the chlorophyll content. The reflective spectrum of the chlorophyll would be covered by that of the anthocyanin from 548 to 760 nm (Moharana & Dutta, 2016; Schlerf et al., 2010). On the other hand, the foliar moisture content of purple corn was 8.6 ± 1.76%. The reflectivity of the moisture is usually presented at 1,080~1,270 nm, which would not influence the reflectivity of the anthocyanin at 570~685 nm (Schlerf et al., 2010). Hence, the presence of chlorophyll and moisture would not affect the model of the foliar anthocyanin content in the current model. However, we would like to try a nonlinear model and compare that with our current model in a future study.

**Figure 2:**
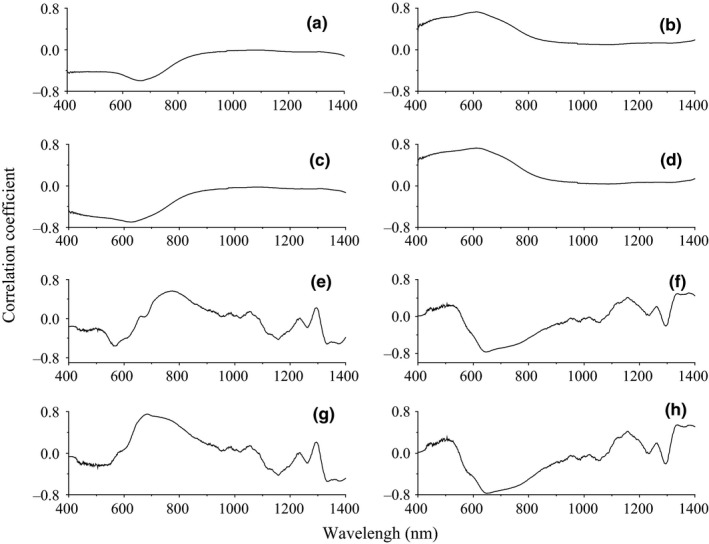
Plots of the correlation coefficient versus *P* (a), 1/*P* (b), lg *P* (c), 1/lg *P* (d), *P*′ (e), ${\left(1/P\right)}^{\prime }$(f), ${\left(\mathrm{lg}P\right)}^{\prime }$ (g), and${\left(1/\mathrm{lg}P\right)}^{\prime }$ (h)

The reflectivity (*P*) of the hyperspectral was inverted to 1/*P*, lg *P*, 1/lg *P*, ${\left(P\right)}^{\prime }$, ${\left(1/P\right)}^{\prime }$, ${\left(\mathrm{lg}P\right)}^{\prime }$, and ${\left(1/\mathrm{lg}P\right)}^{\prime }$. The correlation coefficient between reflectivity and foliar anthocyanin content was plotted against the wavelength of the hyperspectral (Figure 2). The *P* of the hyperspectral was negatively correlated with the foliar anthocyanin content of the purple corn. The correlation coefficient of the visible light band was higher than that of both the near‐infrared and middle‐infrared bands. Specifically, the correlation coefficient in the blue and green band (400~560 nm) was relative stable in the range from −0.41 to −0.43. The maximal absolute value of the correlation coefficient reached 0.60 at 667 nm. The band at 667 nm was the most sensitive band of the plot *P* versus correlation coefficient.

The 1/*P* of the hyperspectral was positively correlated with the foliar anthocyanin content of purple corn. The correlation coefficient of the visible light band was higher than that of both the near‐infrared and middle‐infrared bands. The correlation coefficient of 1/*P* was higher than that of the *P* in the corresponding band. The maximal absolute value of the correlation coefficient reached 0.76 at 607 nm. Consequently, the band at 607 nm was the most sensitive band of the plot 1/*P* versus correlation coefficient.

The lg *P* of the hyperspectral was negatively correlated with the foliar anthocyanin content of purple corn. The correlation coefficient of the visible light band was higher than that of the near‐infrared and middle‐infrared bands. The maximal absolute value of the correlation coefficient reached 0.70 at 626 nm. Consequently, the band of 626 nm was the most sensitive band of the plot lg *P* versus correlation coefficient.

The 1/lg *P* of the hyperspectral was positively correlated with the foliar anthocyanin content of purple corn. The correlation coefficient of the visible light band was higher than that of the near‐infrared and middle‐infrared band. The maximal absolute value of the correlation coefficient reached 0.73 at 613 nm. Consequently, the band of 613 nm was the most sensitive band of the plot 1/lg *P* versus correlation coefficient.

The *P*′ of the hyperspectral was negatively correlated with the foliar anthocyanin content of the purple corn in the 400–670, 1,110–1,250, and 1,330–1,400 nm and was positively correlated with the other bands. The correlation of the visible light band was significantly enhanced compared to that of the transform of the 1/*P*, lg *P*, and 1/lg *P*. The maximal absolute value of the correlation coefficient reached 0.56 at 773 nm. Consequently, the band at 773 nm was the most sensitive band of the plot *P*′ versus correlation coefficient.

The correlation coefficient of the ${\left(1/P\right)}^{\prime }$ was better than that of the nondifferential‐transferred reflectivity. The correlation coefficient of the visible light band was enhanced compared to that of the transform of the lg *P*, and 1/lg *P* and *P*′. The maximal absolute value of the correlation coefficient reached 0.77 at 648 nm. Consequently, the band of 648 nm was the most sensitive band of the plot ${\left(1/P\right)}^{\prime }$ versus correlation coefficient.

The tendency of the correlation coefficient of the ${\left(\mathrm{lg}P\right)}^{\prime }$ was similar to that of *P*′. The correlations of the visible light band, near‐infrared, and middle‐infrared band were all enhanced. Similar to our results, chlorophyll (another plant pigment) is sensitive to the blue band, near‐infrared, and middle‐infrared band (Hunt et al., 2013). The maximal absolute value of the correlation coefficient reached 0.75 at 685 nm. Consequently, the band of 685 nm was the most sensitive band of the plot $\left(\mathrm{lg}P\right)\prime$ versus correlation coefficient.

The tendency of the correlation coefficient of the ${\left(1/\mathrm{lg}P\right)}^{\prime }$’ was similar to that of the ${\left(1/P\right)}^{\prime }$. The correlation of the visible light band, near‐infrared, and middle‐infrared band was in the range of 0.4~0.6. The maximal absolute value of the correlation coefficient reached −0.77 at 648 nm. Consequently, the band of 648 nm was the most sensitive band of the plot ${\left(1/\mathrm{lg}P\right)}^{\prime }$ versus correlation coefficient.

The inversion of the hyperspectral enhanced the correlation coefficient compared to the original reflectivity of the hyperspectral. The band of 570–685 nm was strongly correlated with the foliar anthocyanin content among the band from 400 to 1,400 nm.

### 3.2 Modeling

The hyperspectral model was fitted via linear, polynomial, power, exponential, and logarithmic functions with the sensitive band as the independent variable and the anthocyanin content as the function (Table 1). Each model was randomly trained by a total of 400 samples to ensure its independence. The sensitive bands of the inversions were mainly located in the range of blue, red, and near‐infrared length from 570 to 685 nm. The model fitted via the power function of the inversion ${\left(\mathrm{lg}P\right)}^{\prime }$ showed the highest determination coefficients (0.768) among all hyperspectral models. Hence, the hyperspectral model of “*y* = 3,000,000,000 × *W*_685_^4.5,896^” was used to estimate the foliar anthocyanin content of purple corn.

**Table 1:** Hyperspectral model based on the different inversions

Inversion	Sensitive band (nm)	Hyperspectral model	*R* ^2^
*P*	667	*y *= −0.0009 × *W*_667_ a + 0.0377	.355
		*y *=* *0.00003 × *W*_667_^2^ − 0.0025 × *W*_667_ + 0.0577	.420
		*y *= −0.025 × ln(*W*_667_) + 0.0931	.411
		*y *=* *0.162 × e^−0.124×W667^	.546
		*y *=* *57.038 × *W*_667_^−2.83^	.470
1/*P*	607	*y *=* *0.2753 × *W*_607_ − 0.0116	.527
		*y *= −1.9742 × *W*_607_^2^ + 0.7213 × *W*_607_ − 0.0339	.576
		*y *=* *0.0291 × ln(*W*_607_) + 0.0847	.566
		*y *=* *0.0006 × e^26.43×W607^	.432
		*y *=* *7.8861 × *W*_607_^2.8711^	.490
lg *P*	626	*y *= −0.05 × *W*_626_ + 0.072	.485
		*y *=* *0.0481 × *W*_626_^2^ − 0.1663 × *W*_626_ + 0.1406	.537
		*y *= −0.06 × ln(*W*_626_) + 0.0221	.514
		*y *=* *10.89 × e^−6.313×W626^	.688
		*y *=* *0.0188 × *W*_626_^−7.025^	.634
1/lg *P*	613	*y *=* *0.06 × *W*_613_ − 0.042	.528
		*y *= −0.0145 × *W*_613_^2^ + 0.0884 × *W*_613_ − 0.0556	.530
		*y *=* *0.0561 × ln(*W*_613_) + 0.0187	.519
		*y *=* *0.00001 × e^6.8067×W613^	.604
		*y *=* *0.0127 × *W*_613_^6.7945^	.678
*P*′	570	*y *= −0.6429 × *W*_570_ + 0.031	.274
		*y *=* *16.559 × *W*_570_^2^ − 1.6483 × *W*_570_ + 0.0439	.321
		*y *= −0.017 × ln(*W*_570_) − 0.0494	.311
		*y *=* *0.0463 × e^−69.88×W570^	.287
		*y *=* *0.00001 × *W*_570_^−1.73^	.285
(1/*P*)′	648	*y *= −57.598 × *W*_648_ − 0.0071	.577
		*y *= −85933 × *W*_648_^2^ − 140.12 × *W*_648_ − 0.0235	.648
		*y *=* *0.001 × e^−5359×W648^	.444
(lg *P*)′	685	*y *=* *21.257 × *W*_685_ − 0.0267	.567
		*y *=* *3565.6 × *W*_685_^2^ + 7.3416 × *W*_685_ − 0.0139	.574
		*y *=* *0.0345 × ln(*W*_685_) + 0.2314	.489
		*y *=* *0.00006 × e^2482.8×W685^	.687
		*y *=* *3,000,000,000 × *W*_685_^4.5896^	.768
(1/lg *P*)′	648	*y *= −13.3 × *W*_648_ − 0.0092	.585
		*y *= −453.13 × W_648_^2^ − 15.064 × *W*_648_ − 0.0106	.586
		*y *=* *0.0005 × e^−1529×W648^	.688

a *W*_667_ mean the reflectivity in the hyperspectral band of 667 nm.

### 3.3 Validation of the hyperspectral model

**Figure 3:**
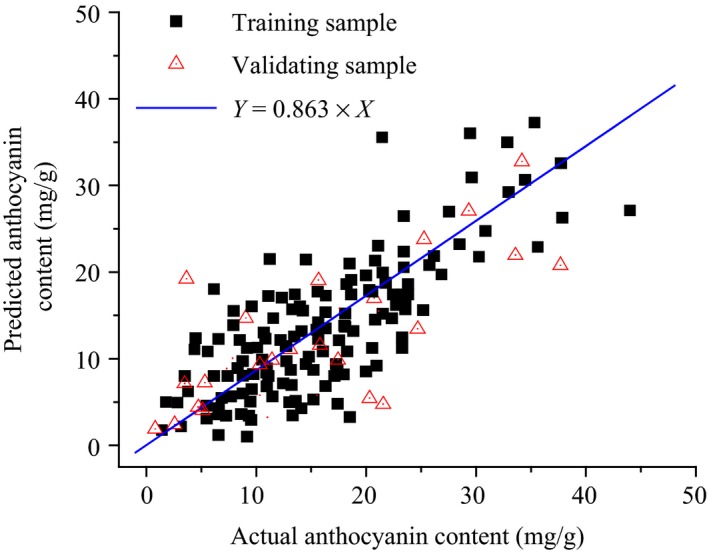
Validation of the hyperspectral model

The hyperspectral model based on the inversion of the ${\left(\mathrm{lg}P\right)}^{\prime }$ was validated via the remaining 100 samples (Figure 3). The RMSE and determination coefficient of the training samples were 0.0065 and 0.932, respectively, while those of the validating sample were 0.0074 and 0.927, respectively. Hence, the foliar anthocyanin content of purple corn was successfully estimated via the hyperspectral model.

Remarkably, the accuracy and stability of the hyperspectral model in the range of 0~20 mg/g were higher than that in the range of 0~40 mg/g. The anthocyanin content of most plants is below 20 mg/g, for example, the blueberry (*Vacciniumspp*) with 10.2 mg/g, the cranberry (*Vacciniumoxycoccus*) with 4.8 mg/g, the mulberry (*Morusnigra*) with 16.1 mg/g, the red currant (*Ribesrubrum*) with 2.5 mg/g, the strawberry (*Fragaria* × *ananassa*) with 5.2 mg/g (Ogawa et al., 2008) (Bechtold, Mahmud‐Ali, & Mussak, 2007), and purple wheat with 9 mg/g (Escribano‐Bailón, Santos‐Buelga, & Rivas‐Gonzalo, 2004). Consequently, the hyperspectral model was further trained with the sample whose foliar anthocyanin content was below 20 mg/g. The RMSE and determination coefficient of the training samples were 0.0047 and 0.954, respectively. Hence, the accuracy and stability of the hyperspectral model were further enhanced when the anthocyanin content of the sample was below 20 mg/g.

## 4 CONCLUSION

The visible light band of purple corn around 650 nm was sensitive to the foliar anthocyanin content. Specifically, the hyperspectral model based on 685 nm fitted via the power function of the inversion ${\left(\mathrm{lg}P\right)}^{\prime }$ showed the highest determination coefficients of 0.768 among all hyperspectral models. The optimum hyperspectral model was validated with the determination coefficient of 0.932 and RMSE of 0.0065. Moreover, the accuracy and stability of the hyperspectral model were further enhanced with a determination coefficient of 0.954 and an RMSE of 0.0047 when the anthocyanin content of the sample was below 20 mg/g. Hence, the hyperspectral model has potential to estimate the foliar anthocyanin content of purple corn or related plants.

## CONFLICT OF INTEREST

All authors have no conflict of interest to declare.
